# A Nurses' Protest

**Published:** 1920-12-04

**Authors:** 


### A NURSES' PROTEST.

A mass meeting of nurses was held at the Cutlers' Hall, Sheffield, on Tuesday, November 23, when Mr. George Slack gave an interesting and instructive address on the Unemployment Bill as it affects nurses. Mr. Sidney Robinson, of the Royal Infirmary Board, presided, and in his opening remarks said the inclusion of nurses was unfair and unnecessary. He said nurses would gain no advantage by their inclusion under the Act. The country would be spending the nurses' money, which was little enough at all times. Mr. Slack outlined the principal points in the Act and dwelt at some length on two main questions, exemption and a special scheme under Section 18 of the Act. He said if the College of Nursing were to fight against inclusion of the nurses, they would have to fight on Section 18, which provides for the preparation of a separate scheme.

Miss Hancox, in answer to a question, said the College of Nursing could not undertake a separate scheme, as it would require the support of at least 50,000 nurses to enable them to work it. Mr. Henry Bedford, Chairman of the Royal Infirmary Board, said he was pleased to be there to support the resolutions which were adopted, and said if Sheffield could give the lead to other cities probably the Ministry of Labour would think better of the matter, and exempt the nursing profession.

Discussion was invited, and many questions were asked. The following resolutions were unanimously adopted :—

"That this meeting, representing the nursing profession of all the Sheffield hospitals and institutions, strongly protest against our inclusion under the provisions of the National Unemployment Act, 1920; and that copies of this resolution be forwarded to the Ministry of Labour, the local Members of Parliament, the Head Office, and all Local Centres of the College of Nursing, the Queen Victoria Jubilee Institute, and the Nurses' Insurance Society."

It was further resolved : "That this large meeting of nurses, representing every hospital and institution in this city, again asks the College of Nursing and the Queen Victoria Jubilee Institute to take steps to prepare and submit a scheme under Section 18 of the Unemployment Act, 1920, failing their getting exemption as per the above-named resolution."

The meeting closed with votes of thanks to the Chairman, Mr. Slack, and others who addressed the meeting.

